# Supplementary material for: No evidence that carotenoid pigments boost either immune or antioxidant defenses in a songbird
Source: Nat Commun. 2018 Feb 5;9:491. doi: 10.1038/s41467-018-02974-x (PMC5799171; doi:10.1038/s41467-018-02974-x)
Supplement: Supplementary file 1 — Supplementary Information [file 41467_2018_2974_MOESM1_ESM.pdf]

SUPPLEMENTARY INFORMATION

No evidence that carotenoid pigments boost either immune or antioxidant defenses in a songbird

Koch et al.

Nature Communications

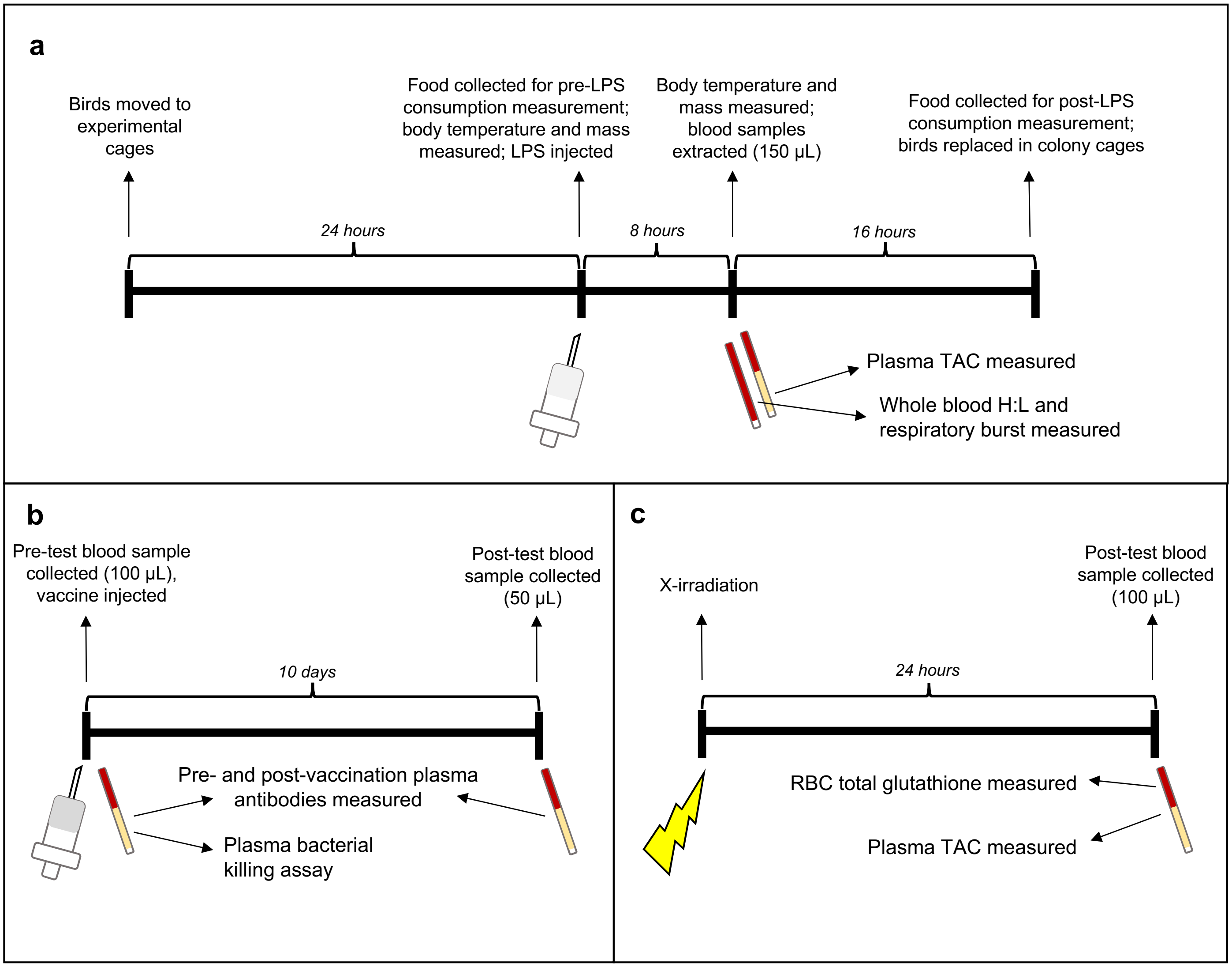

Supplementary Figure 1 Timelines of the three main experimental manipulations and sample collection periods. **a** LPS injection in July-early August 2016, **b** BKA and vaccination in late August 2016, and **c** irradiation in December 2016. At least four weeks elapsed between each experimental segment.

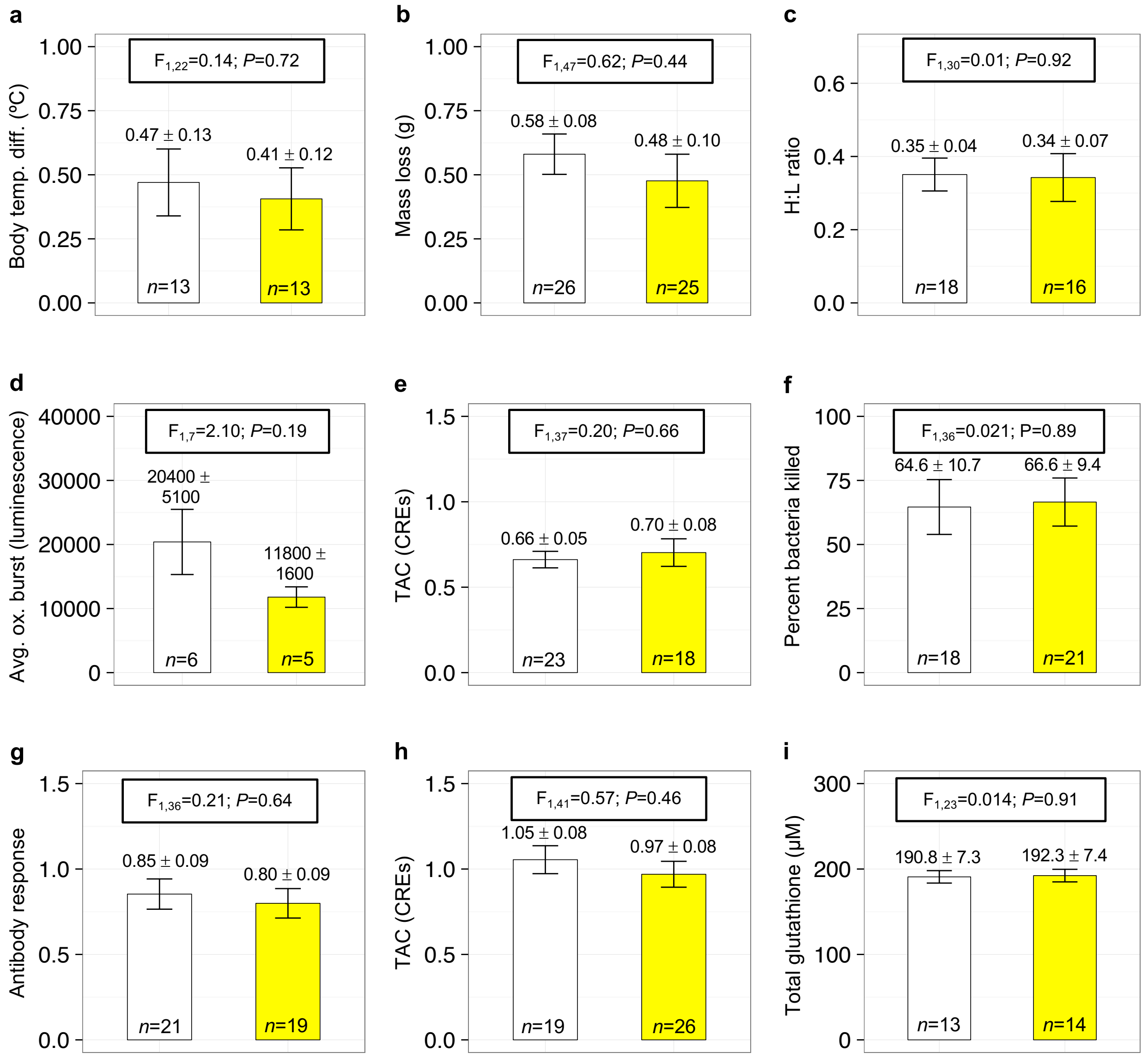

Supplementary Figure 2 Average response (± SEM) of white recessive (WR) and yellow (Y) canaries for a variety of physiological measurement**s. a**, **b** Body temperature (**a**) and mass (**b**) changes in response to LPS, comparing baseline measures to those taken 8 hours after injection. **c** Heterophil to lymphocyte ratio measures on blood smears taken 8 hours after LPS injection. **d** Average oxidative burst response over a one-minute sampling period on red blood cells extracted 8 hours after LPS injection. **e** Total antioxidant capacity in plasma sampled 8 hours after LPS injection. **f** Percent *E. coli* killed in plasma samples relative to positive controls. **g** Average anti-tetanus antibody response (in units of log-transformed milli-optical-density per minute)

measured in plasma samples extracted ten days after tetanus vaccination. **h, i** Total antioxidant capacity (**h**) and total glutathione (**i**) measured in blood samples extracted 24 hours after radiation oxidative challenge. F and *P* values correspond to ANOVA results testing for a significant effect of canary color type (WR vs. Y) on results.

Category	Measurement	Sample sizes	Variable	*F*	*P*
Pre- and post-LPS	Baseline mass	26 WR (16 M, 10 F); 25 Y (17 M, 8 F)	Color	8.92	0.004
			Sex	1.02	0.32
			Color*Sex	0.24	0.63
	Change in mass		Color	0.62	0.44
			Sex	0.01	0.91
			Color*Sex	0.14	0.71
	Baseline food consumption	26 WR (16 M, 10 F); 22 Y (15 M, 6 F)	Color	0.60	0.44
			Sex	3.27	0.08
			Color*Sex	1.23	0.27
	Change in food consumption		Color	2.27	0.14
			Sex	1.84	0.18
			Color*Sex	2.56	0.12
	Baseline body temperature	13 WR (8 M, 5 F); 13 Y (6 F, 7 M)	Color	2.52	0.13
			Sex	0.06	0.81
			Color*Sex	0.17	0.68
	Change in body temperature		Color	0.14	0.72
			Sex	2.46	0.13
			Color*Sex	0.63	0.44
	Total antioxidant capacity	23 WR (14 M, 9 F); 18 Y (13 M, 5 F)	Color	0.20	0.66
			Sex	0.78	0.38
			Color*Sex	0.07	0.79
	Respiratory burst peak luminescence	6 WR (3 M, 3 F); 5 Y (3 M, 2 F)	Color	1.45	0.27
			Sex	0.34	0.58
			Color*Sex	0.09	0.77
	Respiratory burst average luminescence		Color	2.10	0.19
			Sex	1.13	0.32
			Color*Sex	0.45	0.52
	Heterophil:lymphocyte ratio	18 WR (11 M, 7 F); 16 Y (11 M, 5 F)	Color	0.01	0.92
			Sex	0.007	0.94
			Color*Sex	1.01	0.32
Vaccination	Anti-tetanus secondary antibody response	23 WR (13 M, 8 F); 21 Y (13 M, 6 F)	Color	0.21	0.65
			Sex	4.05	0.05
			Color*Sex	2.35	0.13
Bacterial killing assay	Factorial bacterial killing (binomial)	18 WR (9 F, 9 M); 22 Y (15 M, 7 F)	Intercept	z=0.33	0.74
			Color	z=0.007	0.99
			Sex	z=0.99	0.33
			Color*Sex	z=-0.008	0.99
	Percent bacterial killing (ANOVA)		Color	0.02	0.89
			Sex	0.61	0.44
			Color*Sex	5.58	0.024
Post-radiation	Total antioxidant capacity	19 WR (11 M, 8 F); 26 Y (15 M, 11 F)	Color	0.57	0.46
			Sex	0.29	0.59
			Color*Sex	1.64	0.21
	Total glutathione	13 WR (8 M, 5 F); 14 Y (7 M, 7 F)	Color	0.01	0.91
			Sex	5.53	0.03
			Color*Sex	1.60	0.22

Supplementary Table 1 Physiological measurement statistical analysis results. Results of ANOVAs investigating the effects of sex, color, and their interaction on response variables.

Mass, body temperature, and food consumption measurement statistical analyses include tests for whether WR and Y canaries differed in either baseline measurements or in magnitude of measurement change in response to LPS injection. For the bacterial killing assay, results were analyzed both with ANOVA (comparing percent bacteria killed) and with a binomial linear model (grouping results into bins of "fully-killed" or "failed-to-kill"); for the binomial model results, z values are presented in lieu of F statistics.
